# *DNMT1* rs2228611, rs2228612 and *DNMT3A* rs2276598, rs752208 Polymorphisms and Their Association with Breast Cancer Phenotype and Prognosis

**DOI:** 10.3390/medicina60111902

**Published:** 2024-11-20

**Authors:** Meda Marija Kaušylaitė, Justina Jurevičė, Erika Korobeinikova, Jurgita Gudaitienė, Elona Juozaitytė, Rasa Ugenskienė

**Affiliations:** 1 Department of Genetics and Molecular Medicine, Lithuanian University of Health Sciences, LT-50161 Kaunas, Lithuania; rasa.ugenskiene@lsmuni.lt; 2 Oncology Research Laboratory, Institute of Oncology, Lithuanian University of Health Sciences, LT-50161 Kaunas, Lithuania; justina.jurevice@lsmuni.lt; 3 Institute of Oncology, Lithuanian University of Health Sciences, LT-50161 Kaunas, Lithuania; erika.korobeinikova@lsmuni.lt (E.K.); jurgita.gudaitiene@lsmuni.lt (J.G.); elona.juozaityte@lsmuni.lt (E.J.)

**Keywords:** breast cancer, *DNMT1*, *DNMT3A*, polymorphisms, associations, phenotype, survival, prognosis

## Abstract

*Background and Objectives*: Breast cancer is a leading cause of cancer-related deaths globally. This study investigates the impact of genetic polymorphisms in DNA methyltransferases (*DNMT1* and *DNMT3A*) on breast cancer pathomorphology and patient prognosis. Specifically, we focused on *DNMT1* polymorphisms rs2228611 and rs2228612 and *DNMT3A* polymorphisms rs2276598 and rs752208. *Materials and Methods*: Conducted at the Institute of Oncology of the Lithuanian University of Health Sciences, this study included 201 Lithuanian women with early-stage breast cancer. DNA was extracted from peripheral blood samples, and genotyping for the specified polymorphisms was performed using the PCR-RFLP assay. Statistical analyses were applied to evaluate associations between polymorphisms and clinicopathological characteristics. *Results*: The non-carriers of the *DNMT1* rs2228611 G allele were less likely to be diagnosed at an older age, while the *DNMT3A* rs752208 T allele was linked to lower-grade tumors. Survival analysis indicated a potential relationship between *DNMT3A* rs752208 and overall survival, although no significant findings were observed in progression-free or metastasis-free survival. *Conclusions*: This study suggests that the *DNMT1* and *DNMT3A* polymorphisms may influence breast cancer pathomorphology and prognosis. The *DNMT1* rs2228611 G allele may be associated with earlier onset, and the *DNMT3A* rs752208 T allele might correlate with less aggressive tumors. These findings underscore the potential of *DNMT* gene polymorphisms as prognostic biomarkers in breast cancer, warranting further investigation with larger sample sizes.

## 1. Introduction

Breast cancer (BC) ranks among the most commonly diagnosed malignancies and stands as the fifth leading cause of cancer-related fatalities. As per the data provided by GLOBOCAN 2020, an estimated 2.3 million new cases of BC emerged globally [1,2]. Over the past few decades, extensive research, both in basic science and clinical practice, has deepened our understanding of the complex mechanisms driving BC. These efforts have resulted in significant improvements in BC treatment, including advancements in surgery, chemotherapy, radiotherapy, targeted therapies, and immunotherapy [3,4]. The remarkable technological progress notwithstanding, several challenges persist. BC continues to pose a significant global burden due to its high incidence rates, and accurately assessing tumor characteristics—such as aggressiveness and therapeutic response—remains a considerable challenge. The disease’s heterogeneity, along with the unpredictable patterns of metastasis and recurrence, further complicates treatment planning. As a result, predicting overall survival (OS) and disease-free survival (DFS) in BC patients remains imprecise, leading to ongoing concerns within the medical community [3].

One of the mechanisms underlying BC is methylation. This process involves the addition of methyl groups to DNA, which can alter gene expression and contribute to the development and progression of cancer. Generally, DNA methylation is commonly governed by DNA methyltransferases (DNMTs), which facilitate the attachment of a methyl group to the C-5 position of cytosine residues. In humans, three canonical isoforms of DNMTs have been identified: *DNMT1*, *DNMT3A*, and *DNMT3B* [5]. *DNMT3A* and *DNMT3B* are acknowledged as pivotal enzymes involved in the establishment and maintenance of DNA methylation, while DNMT2 and DNMT3L have been experimentally shown to lack the capacity for DNA methylation. *DNMT1*, functioning as the maintenance DNMT, primarily methylates hemimethylated DNA, ensuring the faithful transfer of DNA methylation patterns to daughter strands during replication, while *DNMT3A* and *DNMT3B*, acting as de novo DNMTs, exhibit a preference for unmethylated DNA and are involved in de novo methylation processes during development [6]. Furthermore, DNMT protein levels have been found to correlate with the responses to specific drugs [7].

Gene expression changes are crucial in BC, affecting tumor growth and treatment response. This can result from single-nucleotide polymorphisms (SNPs) in coding genes. SNPs are one of the most common types of genetic variations in the human genome, which can influence genetic susceptibility to cancer when found within genes governing DNA mismatch repair, cell cycle regulation, metabolism, and immunity. From a clinical standpoint, SNPs and their combined effect, known as polygenic risk score (PRS), hold promise as potential diagnostic and therapeutic biomarkers across a wide spectrum of cancer types [8]. When it comes to *DNMT1* and *DNMT3A*, studies have demonstrated elevated expression levels within BC cells [9,10,11,12,13]; therefore, studying them is essential for a better understanding of the disease’s progression and potential therapeutic targets.

SNP rs2228612 induces an amino acid substitution at position 327 in the *DNMT1* protein from isoleucine to phenylalanine, potentially impacting the functionality of *DNMT1* and its role in carcinogenesis, while SNP rs2228611 may influence “the process carcinogenesis by regulating the pattern of alternative splicing of *DNMT1*”. One meta-analysis suggested that the *DNMT1* rs2228612 polymorphism exhibited a significant association with cancer risk in the recessive genetic model and rs2228611 may be associated with BC [14]. Another study showed that individuals with the *DNMT1* rs2228612 GG homozygous genotype demonstrate a reduced risk of developing BC when compared to those with heterozygous or wildtype genotypes [15]. The *DNMT3A* rs227698 polymorphism exhibited an association with tumor response to treatment, which was assessed through the percentage of patients achieving complete or partial remission as per the Response Evaluation Criteria in Solid Tumors classification [16]. In a study of evaluation of *DNMT3A* genetic polymorphisms as outcome predictors in acute myelogenous leukemia (AML), multivariate analysis and combined genotype analysis showed that rs2276598 was associated with increased chemosensitivity (*p* < 0.05), and this SNP was significantly associated with disease prognosis (*p* < 0.05) [17]. For *DNMT3A* rs752208, only one study was found to have investigated this polymorphism, and there is not much information available about it and its impact [18].

Unfortunately, these DNMT gene polymorphisms have not been extensively studied in BC. Therefore, the aim of this study was to identify DNA sequence variation in *DNMT1* rs2228611, rs2228612 and *DNMT3A* rs2276598, rs752208, as well as to investigate their effect on the tumor phenotype and disease prognosis in BC patients.

## 2. Materials and Methods

### 2.1. Study Subject

The study was performed at the Institute of Oncology of the Lithuanian University of Health Sciences. The study received approval from the Kaunas Regional Biomedical Research Ethical Committee (protocols No. BE-2-10 and No. P1-BE-2-10/2014), and written informed consent was obtained from all participants.

This study group included 201 Lithuanian women who had been diagnosed with BC. Patients’ peripheral blood samples were collected between 2010 and 2018 and used for genomic DNA extraction. Clinicopathological information was gathered from medical records in collaboration with oncologists. Patients’ inclusion criteria were as follows: confirmed diagnosis, early BC stage (I–II), and full medical documentation. Meanwhile, patients with the presence of other malignancies, significant comorbidities, poor performance status, and incomplete medical documentation were excluded from the study.

### 2.2. DNA Extraction and Genotyping

Genomic DNA was isolated from peripheral blood leukocytes using a “GeneJet Genomic DNA Purification Kit” (Thermo Fisher Scientific Baltics, Vilnius, Lithuania) as per the manufacturer’s guidelines. Subsequently, the DNA samples were stored at −20 °C until further use.

The genotyping of polymorphisms in the *DNMT1* and *DNMT3A* genes was conducted using the Polymerase Chain Reaction-Restriction Fragment Length Polymorphism (PCR-RFLP) assay. In all cases, the PCR reaction mixture included the components summarized in Table 1. The final PCR volume included 23 μL of mixture and 2 μL of DNA for each sample. Additionally, a negative control was included in each run to monitor potential contamination of the components. All PCR products were fractioned electrophoretically (5 V/cm) on 2 or 3% agarose gel with ethidium bromide to confirm the amplification.

Details regarding the primers, PCR thermal conditions, and product sizes are provided in Table 2.

**Figure 1:**
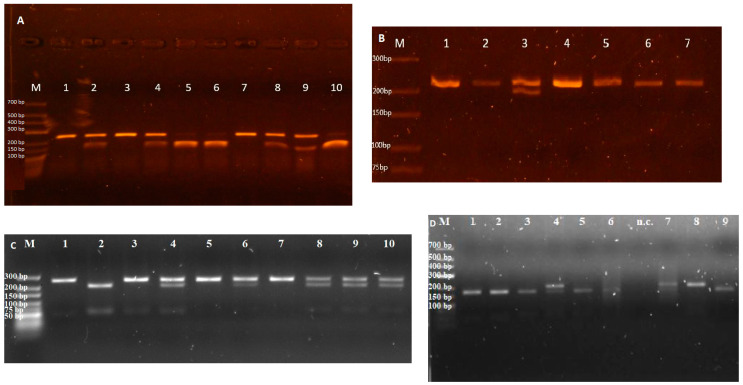
The electrophoresis results of RFLP products. Lane M indicates DNA molecular marker GeneRuler Ultra Low range DNA ladder (**A**,**D**) (Thermo Fisher Scientific Baltics, Lithuania); GeneRuler Low range DNA ladder (**B**,**C**); (**A**) PCR-RFLP products for *DMNT1* (rs2228611). Lanes 1, 3, 7—AA genotype; lanes 2, 4, 8–9—AG genotype; lanes 5–6, 10—GG genotype; (**B**) PCR-RFLP products for *DMNT1* (rs2228612). Lanes 1–2, 4–7—AA genotype; lane 3—AG genotype; (**C**) RFLP products of *DNMT3A* (rs2276598). Lanes 1, 3, 5, and 7 indicate the CC genotype; lanes 4, 6, and 8–10 demonstrate the CT genotype; lane 2 shows the TT genotype; (**D**) RFLP products of *DNMT3A* rs752208. Lanes 1–3, 5–6, and 9 indicate the CC genotype; lanes 4 and 7 demonstrate the CT genotype; lane 8 shows the TT genotype.

Afterwards, the PCR products underwent digestion with specific restriction enzymes: *BsmAI* (for rs2228611), *MspI* (for rs2228612 and rs752208), and *BfaI* (for rs2276598) (Table 3). Incubation of the RFLP reactions was carried out at 37 °C for 2–3 h. Following digestion, the presence of specific alleles was characterized by distinct fragment sizes, as exemplified by the detection of the *DNMT1* rs2228611 G allele, resulting in fragments measuring 124, 111, and 26 base pairs (bp), while the A allele yielded fragments of 235 and 26 bp. For *DNMT1* rs2228612, the A allele resulted in a 219 bp fragment, whereas the G allele produced fragments of 196 and 23 bp. In the case of *DNMT3A* rs2276598, the C allele was represented by 246 bp, while the T allele generated fragments of 194 and 52 bp. The C allele of *DNMT3A* rs752208 was characterized by fragments of 173 and 35 bp, whereas the T allele was represented by a single 208 bp fragment. All RFLP products were fractioned electrophoretically (5 V/cm) on 2 or 3% agarose gel with ethidium bromide (Figure 1).

### 2.3. Statistical Analysis

Statistical analyses were conducted using IBM “SPSS” (Statistical Package for the Social Sciences), version 29.0.2.0, along with Microsoft Excel. The assessment of Hardy–Weinberg equilibrium entailed a comparison of the observed and expected genotype frequencies. The relationships between genotypes, alleles, and tumor characteristics were assessed using Pearson’s Chi-square or Exact tests.

For the odds ratio (OR), an OR >1 was indicated as a positive association, while < 1 suggested a negative association. The confidence interval (CI) for the odds ratio shows the range in which the true OR was likely to fall with 95% confidence. ORs were calculated in both univariate and multivariate logistic regression analyses to examine associations between polymorphisms, tumor characteristics, and disease progression (it is important to note that multivariate analysis was performed only for associations that were statistically significant in univariate analysis). Multivariate models encompassed variables such as age at diagnosis, estrogen receptors (ER), progesterone receptors (PR), human epidermal growth factor receptor 2 (HER2) status, tumor size (T), grade (G), and lymph node involvement (N). Model 1 included the variable of age at diagnosis. Model 2 expanded upon this by adding ER, PR, and HER2 status. Model 3 further included T, G, and N. Statistical significance was determined at *p* < 0.05.

The analysis of progression-free survival (PFS), metastasis-free survival (MFS), and overall survival (OS) involved univariate Cox regression analyses (for statistically significant associations, multivariate Cox regression analysis was conducted). Survival curves were generated using the log-rank test and the Kaplan–Meier method. Statistical significance was determined at *p* < 0.05.

Artificial intelligence (AI) was used in this section.

## 3. Results

### 3.1. Subjects Characteristics

The following information presents the distribution of clinicopathological characteristics of BC patients (*n* = 201). Given that the age at diagnosis data did not follow a normal distribution, the median age was utilized for subsequent analysis. The average age at diagnosis was 48.29 years, while the median age was 47 years, indicating that half of the patients were younger than 47 years at the time of diagnosis (52.2% being ≤47 years and 47.8% being >47 years). The most prevalent subtype was Luminal A (60.2%), followed by Triple-Negative (20.9%), Luminal B (10.9%), and HER2-Positive (8.0%). ER status showed that 68.2% of the patients were positive and 31.8% of the patients were negative. PR status showed that 59.7% were positive and 40.3% of the patients were negative. HER2 status revealed that 81.1% of the patients were negative and 18.9% were positive. Tumor size (T) showed that 64.7% of the patients had T1 (0–2 cm) and 35.3% had T2 (2–5 cm). Lymph node involvement (N) showed that 60.2% were N0 (negative) and 39.8% of the patients were N1 (positive). Differentiation degree (G) revealed that 78.1% of the patients were well to moderately differentiated (G1–G2) and 21.9% were poorly differentiated or undifferentiated (G3–G4). Disease progression was absent in 79.6% and present in 20.4% of the patients. Metastasis was absent in 82.1% of the patients and present in 17.9%. Mortality (death) data showed that 11.9% of the patients were deceased and 88.1% of the patients were alive.

### 3.2. The Distribution of DNMT1 rs2228611, rs2228612, DNMT3A rs2276598, and rs752208 in Patients with BC

In this study, all polymorphisms adhered to Hardy–Weinberg equilibrium (*p* > 0.05) (Supplementary Materials Table S1). The frequency of alleles for *DNMT1* rs2228611 was as follows: 0.447% (A allele) and 0.553% (G allele). The frequency for the *DNMT1* rs2228612 polymorphism was 0.945% (A allele) and 0.055% (G allele). The distribution of *DNMT3A* rs2276598 alleles was as follows: 0.801% (C allele) and 0.199% (T allele); and for *DNMT3A* rs752208, 0.870% (C allele) and 0.130% (T allele).

### 3.3. Association and Logistic Regression Analysis

The statistical analysis was performed to determine the associations between the *DNMT1* rs2228611, rs2228612, *DNMT3A* rs2276598, and rs752208 polymorphisms and the clinicopathological features of BC (n = 201). Statistically significant associations from Pearson’s Chi-square test were as follows: *DNMT1* rs2228611 G allele and age at diagnosis (*p* = 0.029); *DNMT1* rs2228612 and lymph node involvement (*p* = 0.049); *DNMT3A* rs752208 T allele and G3–G4 group (*p* = 0.034); and *DNMT3A* rs752208 and death (*p* = 0.049).

The statistically significant results after univariate logistic regression analysis are summarized in Table 4.

After univariate logistic regression analysis was performed, the results were as follows: for *DNMT3A* rs2228612, the AG genotype, compared to the AA genotype, was associated with a 2.5-fold higher risk of diagnosis at an older age (95% CI: 1.009–6.665, *p* = 0.048) and a 0.301-fold lower risk of lymph node involvement (95% CI: 0.098–0.926, *p* = 0.036). After the multivariate logistic regression analysis, the results were statistically insignificant (95% Cl: 0.900–6.567, *p* = 0.080 and 95% Cl: 0.108–1.180, *p* = 0.091). Carriers of the G allele of *DNMT1* rs2228611 were less likely to be diagnosed at an older age compared to non-carriers (95% CI: 0.234–0.886, *p* = 0.021). The association remained significant for the rs2228611 G allele in model no. 2 (95% CI: 0.299–0.900, *p* = 0.024) and model no. 3 (95% CI: 0.201–0.848 *p* = 0.016) after multivariate logistic regression analysis. For *DNMT3A* rs752208, individuals with the CT genotype compared to the CC genotype (95% CI: 0.073–0.860, *p* = 0.028), as well as carriers of the T allele vs. non-carriers (95% CI: 0.098–0.869, *p* = 0.027), were less likely to have G3–G4 tumors. After the multivariate logistic regression analysis, the association remained significant for the rs752208 T allele in model no. 1 (95% CI: 0.096–0.871, *p* = 0.027), model no. 2 (95% CI: 0.085–0.949, *p* = 0.041), and model no. 3 (95% CI: 0.070–01.863, *p* = 0.029) (Table 5).

More detailed information about univariate logistic regression analysis is covered in Supplementary Materials Table S2.

### 3.4. Survival Analysis

**Figure 2:**
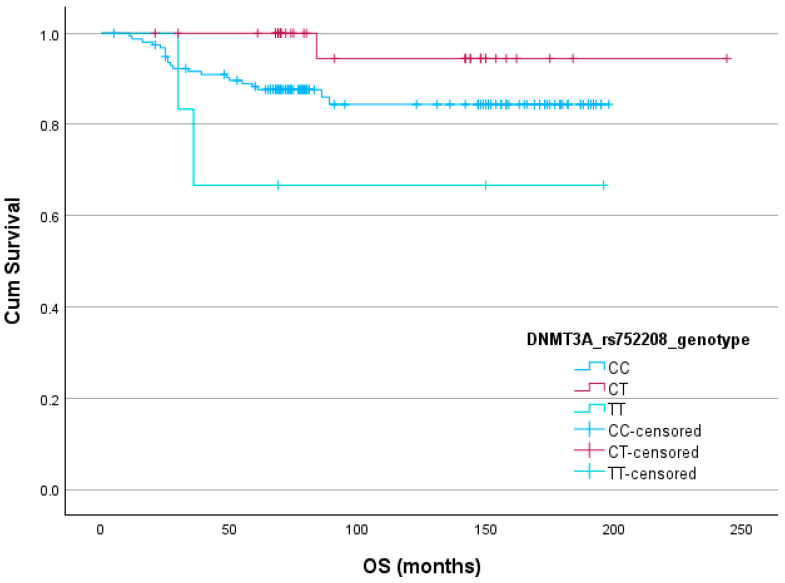
Kaplan–Meier survival curve for the association between *DNMT3A* rs752208 and OS.

Survival analysis of *DNMT1* and *DNMT3A* polymorphisms and PFS, MFS, and OS was performed using the log-rank test. The results showed an association between *DNMT3A* rs752208 and OS (*p* = 0.047) in a genotypic model. None of the tested SNPs were found to be linked with PFS or MFS. The Kaplan–Meier method’s survival curves for statistically significant associations are shown in Figure 2.

Using the univariate Cox regression analysis, no statistically significant associations between the studied polymorphisms and PFS, MFS, or OS were determined (Supplementary Materials Table S3). Therefore, the multivariate Cox regression analysis was not performed.

## 4. Discussion

To our knowledge, the associations between *DNMT1* (rs2228611, rs2228612), *DNMT3A* (rs2276598, rs752208) polymorphisms and BC clinicopathological features and prognoses in the Lithuanian population have not yet been studied. We identified statistically significant associations between *DNMT1* rs228611, 0 *DNMT3A* rs752208, and the studied characteristics. However, no associations were found for *DNMT3A* rs2276598, and the associations of *DNMT1* rs2228612 lost significance in the multivariate model.

The first polymorphism to be analyzed, along with its associations with BC clinicopathological features, was *DNMT1* rs2228611. In our study, this polymorphism showed a significant association between its G allele and age at diagnosis, meaning that the presence of the G allele may influence the age when the disease is detected or becomes clinically apparent. There were no significant associations between rs2228611 and OS, PFS and MFS. Xiang et al. reported that the AG genotype in rs2228611 had a higher frequency in patients than in controls (*p* = 0.015). Therefore, it is likely that the rs2228611 AG genotype may carry a potential risk for invasive ductal carcinoma (IDC) development in the breast. However, significant associations between *DNMT1* SNPs and lymph node metastasis, tumor size, and ER status in breast IDC cases were not found [21]. In a Polish study, the impact of DNMT gene variants on the risk of ovarian cancer was examined. The findings revealed that the *DNMT1* rs2228611 polymorphism is associated with an increased risk of ovarian cancer in Polish women (OR 1.836 (1.143–2.949), *p* = 0.0114, pcorr = 0.0342). However, *DNMT1* rs2228611 did not present a significant association with ovarian cancer development in either dominant or recessive inheritance models. The effect on the age of diagnosis was also investigated, but no significant correlations were found [22]. The results of these studies support our data.

In our study, we also investigated associations of *DNMT1* rs2228612 and BC parameters. However, no statistically significant relationship between this SNP and BC features or patients’ survival was found. In contrast to our findings, Chinese study demonstrated BC susceptibility in women with the GG homozygote genotype [23]. Another study focusing on the central European Caucasian female population was able to demonstrate a significant association between rs2228612 and the risk of BC in the population (*p* = 0.030), GG homozygous genotype (variant) have a lower risk of developing breast cancer compared to heterozygous or wildtype genotypes. Nevertheless, the analysis of genotyping results in relation to clinical parameters, including age at diagnosis, disease duration, histology, TNM status, ER status, PR status, HER2 status, chemotherapy response, and survival, revealed no statistically significant correlations [15]. In a study conducted by Chang SC et al., the relationship between *DNMT1* polymorphism and the development of esophagus, stomach, and liver cancer in a Chinese population was examined. The researchers found an inverse association between esophageal cancer and the G allele of the *DNMT1* rs2228612 polymorphism [24]. Even though we did not find any significant associations between this SNP and BC clinicopathological features, the findings discussed above suggest that rs2228612 may still play a potentially deleterious role in cancer development.

In the case of *DNMT3A* rs2276598, no statistically significant results were determined in our study. However, only a limited number of researchers have aimed to analyze the contribution of this specific polymorphism to the pathomorphological parameters of breast tumors and the course of the disease. The investigation conducted by A. Puccini et al. suggests a strong relationship between *DNMT3A* rs2276598 and tumor response to treatment, which was defined as the percentage of patients achieving either complete or partial remission according to the Response Evaluation Criteria in Solid Tumors classification [16]. Yuan et al. reported on rs2276598 in acute myelogenous leukemia (AML) as an outcome predictor. Multivariate analysis and combined genotype analysis showed that rs2276598 was associated with increased chemosensitivity (*p* < 0.050) and was significantly associated with disease prognosis (*p* < 0.050) [17].

In the present study, after multivariate logistic regression analysis, we determined statistically significant associations between *DNMT3A* rs752208 T allele and a differentiation degree (G group). Our findings suggest that the T allele may influence the aggressiveness and differentiation status of the tumor, potentially making it a useful marker for predicting tumor grade. For example, the T allele might be associated with more poorly differentiated, aggressive tumors. Association with OS indicated that the *DNMT3A* rs752208 polymorphism affected survival outcomes; however, this was not confirmed in Cox regression analyses. A comprehensive literature review revealed that there are no studies directly supporting our research results.

There are several limitations in this study. In the case of the *DNMT1* rs2228612 polymorphism, only the genotypic model was analyzed, as there were no patients in one of the groups (for the GG genotype). Another limitation of this study is the small sample size; therefore, it would be beneficial to continue this research with a larger sample cohort.

## 5. Conclusions

To conclude, we identified that carriers of the G allele vs. non-carriers of *DNMT1* rs2228611 were less likely to be diagnosed at an older age. Additionally, the individuals with the *DNMT3A* rs752208 CT genotype vs. the CC genotype were less likely to have G3-G4 tumors. However, further detailed studies involving a larger cohort are recommended to confirm our findings.

## Figures and Tables

**Table 1:** Protocol for PCR mixture.

Reagent	Volume for 1 Sample (μL)
Distilled water (dH_2_O)	16.91
10× DreamTaq Buffer	2.5
Forward primer (20 pmol/μL)	0.38
Reverse primer (20 pmol/μL)	0.38
DreamTaq DNA polymerase (5 U/μL)	0.13
dNTP mix (25 mM)	0.2
MgCl_2_ (25 mM)	2.5

**Table 2:** Primer sequences, PCR thermal conditions, and product sizes.

Gene, SNP	Primers Sequences	Annealing Temperature (°C)	Cycles of PCR	Size of PCR Product
*DNMT1* rs2228611 ^1^		63	35	235 bp
forward primer: reverse primer:	5′-GTACTGTAAGCACGGTCACCTG-3′ 5′-TATGTTGTCCAGGCTCGTCTC-3′			
*DNMT1* rs2228612 ^2^		52	35	219 bp
forward primer: reverse primer:	5′-AGAACCTGAAAAAGTAAATCCACCG-3′ 5′-CATGTGATTCACCCGCTTCAG-3′			
*DNMT3A* rs2276598 ^3^		57.6	40	246 bp
forward primer: reverse primer:	5′-TAGCCAACCAACAGAGAGCA-3′, 5′- CATGATTGAATGGGCCCTGG-3′			
*DNMT3A* rs752208 ^4^		57.6	40	208 bp
forward primer: reverse primer:	5′-TCTTGAGTCGGGTGTGTCAG-3′, 5′-CCCTTCCTACTACTGACGCC-3′			

Primer sequences were described by Yıldız et al. [19] ^1^ and Arakawa et al. [20] ^2^; primers for *DNMT3A* polymorphisms (rs2276598 and rs752208) were developed on site using various computer programs ^3,4^.

**Table 3:** Protocol for RFLP mixture.

Reagent	Volume for 1 Sample (μL)
Distilled water (dH_2_O)	2.75
10× Buffer Tango	1.5
Restriction Enzymes (10 U/μL) ^1^	0.75

*Alw26I* (*BsmAI*) for rs2228611, *MspI* (*HpaII*) for rs2228612 and rs752208, and BfaI (FspBI) for rs2276598 ^1^.

**Table 4:** The statistically significant associations between genotypes or alleles and clinicopathological features after univariate logistic regression analysis (*n* = 201).

Gene	SNP	Genotype or Allele	Feature	OR	95% CI	*p*
*DNMT1*	rs2228612	AG versus AA (ref.)	>50 years at the time of diagnosis	2.593	1.009–6.665	0.048
		AG versus AA (ref.)	Positive lymph node involvement	0.301	0.098–0.926	0.036
	rs2228611	AG+GG versus AA	>50 years at the time of diagnosis	0.455	0.234–0.886	0.021
*DNMT3A*	rs752208	CT versus CC (ref.)	G3–G4 group	0.251	0.073–0.860	0.028
	rs752208	CT+TT versus CC	G3–G4 group	0.293	0.098–0.869	0.027

The abbreviations in the table: SNP—single-nucleotide polymorphism; *p*—*p*-value, representing the statistical significance of the association (*p*-value < 0.05 is generally considered statistically significant); G3–G4—higher-grade tumors; ref. stands for “reference” and refers to the baseline or comparison genotype or allele in a study.

**Table 5:** Multivariate logistic regression analysis (*n* = 201).

Dependent	Gene, SNP	Covariates	Odds	Cl 95%	*p* (sig.)	Odds	Cl 95%	*p* (sig.)	Odds	Cl 95%	*p* (sig.)
Age at diagnosis	*DNMT1* rs2228612	AG genotype vs. AA genotype				2.962	1.110–7.906	0.030	2.431	0.900–6.567	0.080
		ER+ vs. ER−				2.535	1.110–7.906	0.032	2.987	1.205–7.407	0.0018
		PR+ vs. PR−				1.245	0.563–2.751	0.588	0.908	0.379–2.177	0.829
		HER2+ vs. HER2−				1.154	0.544–2.452	0.709	1.097	0.495–2.431	0.820
		T3–T4 vs. T1–T2							1.426	0.721–2.821	0.308
		N1 vs. N0							0.370	0.187–0.731	0.004
		G3–G4 vs. G1–G2							0.370	0.228–1.243	0.145
	*DNMT1* rs2228611	AG+GG versus AA				0.454	0.229–0.900	0.024	0.413	0.201–0.848	0.016
		ER+ vs. ER−				2.450	1.049–5.722	0.038	3.044	1.227–7.552	0.016
		PR+ vs. PR−				1.196	0.541–2.644	0.659	0.833	0.344–2.016	0.686
		HER2+ vs. HER2−				1.068	0.500–2.284	0.865	1.039	0.466–2.317	0.926
		T3–T4 vs. T1–T2							1.337	0.674–2.652	0.406
		N1 vs. N0							0.329	0.166–0.650	0.001
		G3–G4 vs. G1–G2							0.521	0.221–1.230	0.137
Lymph node involvement	*DNMT1* rs2228612	AG genotype vs. AA genotype	0.351	0.112–1.097	0.072	0.365	0.116–1.153	0.086	0.357	0.108–1.180	0.091
		Age *	0.449	0.249–0.809	0.008	0.399	0.214–0.746	0.004	0.375	0.191–0.735	0.004
		ER+ vs. ER−				3.187	1.274–7.973	0.013	3.036	1.153–7.992	0.025
		PR+ vs. PR−				0.414	0.177–0.965	0.041	0.637	0.254–1.599	0.337
		HER2+ vs. HER2−				1.465	0.677–3.170	0.333	1.648	0.711–3.820	0.244
		T3–T4 vs. T1–T2							4.255	2.191–8.261	0.000
		G3–G4 vs. G1–G2							1.765	0.737–4.225	0.202
G group	*DNMT3A* rs752208	CT genotype vs. CC genotype	0.248	0.071–0.860	0.028	0.287	0.074–1.107	0.070	0.274	0.069	0.066
		Age *	0.371	0.179–0.769	0.008	0.536	0.234–1.227	0.140	0.665	0.273–1.617	0.368
		ER+ vs. ER−				0.661	0.235–1.857	0.432	0.451	0.152–1.338	0.151
		PR+ vs. PR−				0.116	0.039–0.344	0.000	0.140	0.047–0.418	0.000
		HER2+ vs. HER2−				0.212	0.069–0.654	0.007	0.167	0.052–0.542	0.003
		T3–T4 vs. T1–T2							1.733	0.725–4.145	0.216
		N1 vs. N0							2.081	0.834–5.192	0.116
	*DNMT3A* rs752208	CT+TT versus CC	0.290	0.096–0.871	0.027	0.283	0.085–0.949	0.041	0.245	0.070–0.863	0.029
		Age *	0.371	0.179–0.769	0.008	0.536	0.234–1.227	0.140	0.664	0.273–1.615	0.367
		ER+ vs. ER−				0.662	0.236–1.856	0.433	0.457	0.154–1.355	0.158
		PR+ vs. PR−				0.117	0.039–0.345	0.000	0.141	0.047–0.422	0.000
		HER2+ vs. HER2–				0.212	0.069–0.654	0.007	0.169	0.052–0.547	0.003
		T3–T4 vs. T1–T2							1.711	0.719–4.075	0.225
		N1 vs. N0							2.062	0.829–5.124	0.119

The abbreviations in the table: Age *—older age at diagnosis (>47) versus early at diagnosis (≤47), vs—versus, ER—estrogen receptor, PR—progesterone receptor, HER2—human epidermal growth factor receptor 2. Tumor staging is indicated by T3–T4 versus T1–T2, where T1–T2 represents smaller tumors and T3–T4 indicates larger or more invasive tumors. N1 versus N0 refers to nodal involvement, with N1 indicating node involvement and N0 indicating no nodal involvement. Tumor grade is shown as G3–G4 versus G1–G2, where G1–G2 represents lower-grade tumors and G3–G4 higher-grade tumors.

## Data Availability

The data presented in this study are available from the corresponding author upon reasonable request.

## Supplementary Materials

The following supporting information can be downloaded at: https://www.mdpi.com/article/10.3390/medicina60111902/s1, Table S1: The distribution of DNMT1 rs2228611, rs2228612, DNMT3A rs2276598, and rs752208 in patients with BC (n = 201); Table S2: Univariate logistic regression analysis (n = 201); Table S3: Univariate Cox regression analysis (n = 201).
